# Comparison of the effect of temperature and water potential on the seed germination of five *Pedicularis kansuensis* populations from the Qinghai–Tibet plateau

**DOI:** 10.3389/fpls.2022.1052954

**Published:** 2022-11-24

**Authors:** Gensheng Bao, Peng Zhang, XiaoXing Wei, Yongchao Zhang, Wenhui Liu

**Affiliations:** ^1^ Key Laboratory of Qinghai-Tibetan Plateau Forage Germplasm Research, Qinghai Academy of Animal and Veterinary Medicine, Xining, China; ^2^ State Key Laboratory of Sanjiangyuan Ecology and Plateau Agriculture and Animal Husbandry, Qinghai University, Xining, China; ^3^ Qinghai University, Xining, China

**Keywords:** seed germination, temperature, water potential, *Pedicularis kansuensis*, thermal time model, hydrotime model, Qinghai-Tibetan plateau

## Abstract

Temperature and water potentials are considered the most critical environmental factors in seed germinability and subsequent seedling establishment. The thermal and water requirements for germination are species-specific and vary with the environment in which seeds mature from the maternal plants. *Pedicularis kansuensis* is a root hemiparasitic weed that grows extensively in the Qinghai–Tibet Plateau’s degraded grasslands and has seriously harmed the grasslands ecosystem and its utilization. Information about temperatures and water thresholds in *P*. *kansuensis* seed germination among different populations is useful to predicting and managing the weed’s distribution in degraded grasslands. The present study evaluated the effects of temperature and water potentials on *P*. *kansuensis* seed germination in cool and warm habitats, based on thermal time and hydrotime models. The results indicate that seeds from cool habitats have a higher base temperature than those from warm habitats, while there is no detectable difference in optimum and ceiling temperatures between habitats. Seed germination in response to water potential differed among the five studied populations. There was a negative correlation between the seed populations’ base water potential for 50% (*Ψ*_b(50)_) germination and their hydrotime constant (*θ*_H_). The thermal time and hydrotime models were good predictors of five populations’ germination time in response to temperature and water potentials. Consequently, future studies should consider the effects of maternal environmental conditions on seed germination when seeking effective strategies for controlling hemiparasitic weeds in alpine regions.

## Introduction

The environment in which seeds mature from a maternal plant plays a critical role in regulating germination and determining the destiny of seedlings (Fenner and Thompson, 2005; Baskin and Baskin, 2014). Water and temperature are considered the most essential environmental factors in the germinability of seeds (Bradford, 1999). Information about seed germination responses to these two environmental factors can help explain why a species’ germinability differs in different habitats (Donohue et al., 2005; Hu et al., 2015; Huang et al., 2016; Zhang et al., 2020).

Temperature regulates seed germination, seedling recruitment, and population distribution (Baskin and Baskin, 2014; Maleki et al., 2022). The seed germination of many plant species is triggered by temperature when seed dormancy is broken (Fenner and Thompson, 2005). Three cardinal temperatures, namely minimum, optimum, and maximum, are widely used to define the temperature range for seed germination (Trudgill et al., 2000). The minimum/base (*T*_b_) and maximum/ceiling (*T*_c_) temperature are considered the threshold temperatures for seed germination. Consequently, germination does not occur when temperatures are lower or higher than *T*_b_ and *T*_c_, respectively. By contrast, seed germination is most rapid at the optimum temperature (*T*_o_) (Alvarado and Bradford, 2002; Bradford, 1999). Empirical studies have revealed that the cardinal temperatures for seed germination differ significantly between plant species originating from the same or different natural habitats (Tudela-Isanta et al., 2018; Fernández-Pascual et al., 2020). For instance, some studies found that cardinal temperatures differ for *Banksia*, *Stipa*, Fabaceae, Asteraceae and Poaceae seeds collected from populations located in different ecosystems (Imbert et al., 1999; Cochrane et al., 2014; Hu et al., 2015; Zhang et al., 2020). These findings reflect, in part, the plant species’ ecological adaptation to the habitat’s environmental conditions, as well as predicting their geographical distribution ranges (Alvarado and Bradford, 2002; Dürr et al., 2015). The thermal time approach has been extensively and successfully applied to model the germination rate of plant seeds (Alvarado and Bradford, 2002; Onofri et al., 2018). The thermal time model may be more effective than calendar dates in predicting germination time when the designed temperatures are outside the experimental data range (Bradford, 1999). Compared to the studies predicting the cardinal temperatures for species from different habitats, few studies have used the thermal model to estimate the thermal requirements for seed germination of a single species originating from different ecosystems.

Water availability is another essential environmental factor in seed dormancy and germination time (Baskin and Baskin, 2014; Dantas et al., 2020; Zhang et al., 2020). Bradford (1999) found that non-dormant seeds could germinate after accumulating sufficient thermal time at a suitable water potential. To demonstrate the effects of decreased water potential on the progress of seed germination, Bradford (1999) formulated a hydrotime model, this model is used to estimate germination rates at different water potentials in a manner analogous to the thermal time model. The response of seed germination to water potentials is species-specific, suggesting that different species have different hydrotime constants (*θ*_H_), and thresholds or base water potentials (*ψ*_b_) (Baskin and Baskin, 2014; Yi et al., 2019). Seeds with low *ψ*_b_ germinate better at low water potentials than seeds with high *ψ*_b_ (Zhang et al., 2020). Furthermore, the base water potential at which seed germination differs within or among plant species originates from different geographical habitats (Imbert et al., 1999; Hu et al., 2015; Lima and Meiado, 2017; Zhang et al., 2017; Zhang et al., 2020). The germination of species adapted to humid environments may be more sensitive to water stress than that of species adapted to arid environments (Gao et al., 2021). For example, Hu et al. (2015) and Zhang et al. (2020) found that the base water potential requirements for the germination of Fabaceae and *Stipa* species collected from dry and wet habitats differed significantly, but not between habitats. Few studies have reported that seeds from different populations of the same species respond differently to water stress in the seed germination stage (Imbert et al., 1999; Baskin and Baskin, 2014). One example is *Pinus brutia* (originating from three geographical areas), whose seed germination differs in sensitivity to moisture stress (Tilki and Dirik, 2007). Furthermore, Zhang et al. (2020) found that *Stipa bungeana* seeds differ among populations in their sensitivity to water stress. However, few comparative studies have been conducted using the hydrotime model to estimate the threshold water requirements for the seed germination of a single species collected from different populations.

*Pedicularis kansuensis* is an annual or biennial root hemiparasitic weed that can acquire some water, nutrients, and carbon compounds from its neighboring hosts through parasite-derived structures called haustoria (Bao et al., 2015a). This weed is extensively distributed in the Qinghai–Tibet Plateau’s degraded grasslands through the combined effects of climate change, high seed production, and unpalatability to herbivores (Bao et al., 2015b; Sui et al., 2016; Wang et al., 2019; Wei et al., 2019; Wei et al., 2020; Wei et al., 2022). *P*. *kansuensis*’s extensive spread has had serious effects on grassland utilization because the hemiparasite suppresses the growth of grasses and legumes (Bao et al., 2015b; Qin et al., 2022; Sui et al., 2022). A high seed-setting rate, effective seed dispersal capability, and rapid adaptation to new habitats can accelerate the speed of weed spread (Javaid et al., 2022). Therefore, seed germination is considered the most critical phase in plant development and the establishment of a new population (Koger et al., 2004). Previous studies have shown that temperature and water potentials are the most important factors in *P*. *kansuensis* seed germination and seedling establishment (Wang et al., 2008; Hu et al., 2022). However, these studies overlooked critical information regarding the temperature threshold (i.e., the three cardinal temperatures) and the base water potential for *P*. *kansuensis* seed germination. Moreover, their results regarding the effects of temperature and water potentials on seed germination were partly contradictory, as *P*. *kansuensis* seeds were collected from a single population.

The present study aimed to quantify the germination of *P*. *kansuensis* seeds from five populations under different temperatures and water potentials and to test the relationship between germination and temperature or water potential using the thermal time and hydrotime models, respectively. The following was hypothesized: (1) The three cardinal temperatures (*T*_b_, *T*_o_, and *T*_c_) for *P*. *kansuensis* seed germination in habitats with low annual temperatures are higher than those in habitats with high annual temperatures, and (2) *P*. *kansuensis* seeds collected from habitats with high precipitation are more sensitive to water stress than those from habitats with relatively low precipitation.

## Materials and methods

### Seed collection

In October 2020, mature *P*. *kansuensis* seeds were collected from five natural populations on the Qinghai–Tibet Plateau (**Table 1**). Data on the collecting sites’ mean annual temperatures and precipitation were obtained from nearby weather stations. According to Hu et al.’s (2015) and Zhang et al.’s (2020) methods, seeds originating from the Tibet Plateau (SN and LZ) and the Qinghai Plateau (YS, GL, and HB) were classified into cool and warm habitats, respectively, as the habitats’ mean annual precipitation and temperatures were distinctly different (**Table 1**). As the mature seeds were stored in capsules before dehiscence (Sui et al., 2016), several hundred individuals containing indehiscent capsules were collected from each of the five collection sites and taken to the laboratory. The seeds were gently shaken out of the cracked capsules. Shedding seeds were cleaned using a sieve to remove the residues. As *P*. *kansuensis* seeds have non-deep physiological dormancy (Hu et al., 2022), and previous studies have suggested that dry storage at room temperature is an effective method for breaking this dormancy type (Liu et al., 2011; Baskin and Baskin, 2014). Therefore, the seeds were stored in an envelope at room temperature (20%–30% relative humidity, 10−20°C) to after-ripen until germination tests were conducted.

**Table 1:** Origin of the *Pedicularis kansuensis* plant lineages and their attributes.

Habitat	Collection site (population code)	Habitat	Latitude	Longitude and latitude	Mean annual precipitation (mm)	Mean annual temperature (°C)
Warm	Lazi (LZ)	Alpine meadow	4004 m	29°05′14″ (N) 87°39′12″ (E)	328	7.37
	Shannan (SN)	Alpine meadow	4660 m	28°53′36″ (N) 90°18′05″ (E)	385	9.80
Cool	Batang (YS)	Alpine meadow	3860 m	32°50′28″ (N) 97°05′28″ (E)	482	1.57
	Sanjiaocheng (HB)	Alpine steppe	3258 m	37°17′30″ (N) 100°12′13″ (E)	424	0.41
	Yinmatan (GL)	Wetland	4217 m	34°40′14″ (N) 98°02′43″ (E)	586	−2.43

The seed size (including length and width) of 50 seeds originating from different populations was measured, and their thousand seed weight was measured in five sub-samples of the five seed origins. The thousand seed weight was determined in accordance with the International Rules of Seed Testing (ISTA, 2009).

### Effect of temperature on seed germination

In March 2021, five replicates of 50 seeds originating from different populations were placed on two sheets of filter paper (Jiaojie, Fushun, China) in 9-cm-diameter Petri dishes and moistened with 6 mL of distilled water. The seeds were incubated in an incubator at 10, 15, 20, 25, 30, and 35°C (Top Instrument, incubator model RTOP−260Y), with a 12 h light (mean photon flux density of 60 μmol·m^-2^·s^-1^, 400−700 nm) and 12 h dark diurnal cycle. The number of germinated seeds was examined under white fluorescent light and monitored every 6, 12, 18, and 24 h until no further germination was observed within three days. The criterion for germination was the length of the radicle reaching at least 2 mm. The germinated seeds were removed from the Petri dishes. To avoid the variance caused by incubator conditions, each incubator’s temperature, humidity, and light were monitored daily, and their Petri dishes were randomly rearranged every second day.

### Effect of water potential on seed germination

The seeds’ germination responses to water potential were determined by incubating the seeds in light at 20°C, with a water potential of 0, −0.2, −0.4, and −0.6 MPa. The germination substrates’ water potential was determined using polyethylene glycol 6000 (PEG) solutions, which were prepared according to Michel and Kaufmann’s (1973) methods. For each treatment, five replicates of 50 seeds were planted in 9-cm-diameter Petri dishes on two layers of filter paper and moistened with 5 mL of distilled water (control) or different concentrations of PEG solution. The Petri dishes were sealed in Parafilm to reduce evaporation. To ensure relatively constant water potential during the germination period, the seeds were transferred to a new filter paper with fresh solution or distilled water every two days. The number of geminated seeds was recorded every 6, 12, 18, and 24 h until no further germination was observed within three days. Seeds with a radicle length exceeding 2 mm were considered germinated.

### Statistical analysis

A two-way analysis of variance (ANOVA) was used to examine the effects of temperature or water potential, seed populations, and their interactive effects of seed populations and temperature or water potential on germination percentage and rate (1/*T*_50_). The significant difference in seed germination percentage, germination rate, cardinal temperature (*T*_b_, *T*_o_, and *T*_c_), and base median water potential [*Ψ*_b(50)_] from the same origin under different temperatures or water potentials was tested with a *post-hoc* test with multiple comparisons of the means, following a one-way ANOVA. The germination percentage data were standardized and transformed using arcsine transformation to meet the assumption of a normal distribution and homogeneity before analysis. All data were processed with IBM Statistical Product and Service Solution (SPSS) software (version 19.0, Shanghai, China). For a given temperature or water potential, the cumulative germination percentage for each replicate of seeds from different collecting sites was probit-transformed and regressed against time, and the time for cumulative germination (*t*_g_) to reach different percentiles (20%−80%) was estimated by the function described by Steinmaus et al. (2000).

The temperature range for seed germination was separated into suboptimal and supraoptimal temperatures in the thermal time analysis, and the temperature with the highest germination rate (1/*T*_50_) was considered the demarcation of the sub- and supraoptimal temperature ranges. Each subpopulation’s germination rate (germination percentage 20%−80%) was regressed against the temperatures. Linear models (Eqs. 1 and 2) were employed to estimate the cardinal temperature (*T*_b_, *T*_o_, and *T*_c_), as suggested by Hu et al. (2015).


(Eq. 1) $$1/t_{g}=(T-T_{b})/\theta_{1}$$

(Eq. 2) $$1/t_{g}=(T_{c}-T)/\theta_{2}$$

where *t*_g_ is the actual time to germination for a given percentage, *T* is the actual germination temperature, *T*_b_ is the base temperature, below which seeds will not germinate, *T*_c_ is the ceiling temperature, above which seeds will also not germinate, and *θ*_1_ and *θ*_2_ are the thermal times of the suboptimal or supraoptimal temperature, respectively.

For the thermal time model construction in the suboptimal or supraoptimal temperature ranges, the cumulative germination values [probit (*g*)] from all monitoring times and suboptimal or supraoptimal temperatures were pooled and regressed against a function of time (*t*_g_) and temperature (*T*), according to Eqs. 3 and 4 (see Hu et al., 2015).


(Eq. 3) $$probit(g)=[log(T-T_{b})t_{g}-log(\theta_{T(50)})]/\sigma_{\theta T}$$

(Eq. 4), $$probit(g)=[T+(\theta_{T}/t_{g})-T_{c(50)}]/\sigma_{Tc}$$

where *θ*_T_ is the thermal time constant for all individual seeds, *σ*_Tc_ is the standard deviation among individual seeds in the population or the inverse of the slope of the probit regression line, *T_c_*_(50)_ is the median temperature for germination, and the value of *T_c_*_(50)_ can be calculated according to the regression of time when *g* = 50% or probit (*g*) = 0.

The hydrotime models (Eqs. 5 and 6) were used to estimate the hydrotime constant *θ*_H_ (MPa−days) and the base water potential *Ψ*_b_(g) (MPa), as described by previous studies (Bradford, 1999).


(Eq. 5) $$\theta_{H}=[\psi -\psi_{b}(g)]t_{g}$$

(Eq. 6) $$probit(g)=[\psi -(\theta_{H}/t_{g})-\psi_{b(50)}]/\sigma_{\psi b}$$

## Results

The seed size and the thousand seed weight of *P*. *kansuensis* plant lineages originating from HB were found to be the lowest (**Table 2**). The seed size and thousand seed weight did not differ among the other seed origins (**Tables 2**, **S1**).

**Table 2:** Seed size (length, width) and thousand seed weight of *P. kansuensis* collected from warm and cool habitats.

Habitat	Collecting site	Length (mm)	Width (mm)	Thousand seed weight (g)
Warm	LZ	2.228 ± 0.030a	1.092 ± 0.016a	0.807 ± 0.011b
	SN	2.104 ± 0.024b	0.970 ± 0.014b	0.715 ± 0.008c
Cool	YS	2.268 ± 0.025a	1.113 ± 0.018a	0.901 ± 0.014a
	HB	1.876 ± 0.024c	0.924 ± 0.019c	0.524 ± 0.007d
	GL	2.241 ± 0.038a	1.098 ± 0.021a	0.795 ± 0.015b

The different lowercase letters indicate the significant differences (P < 0.05) in the seed size and thousand seed weight of *P. kansuensis* from different origins.

**Figure 1:**
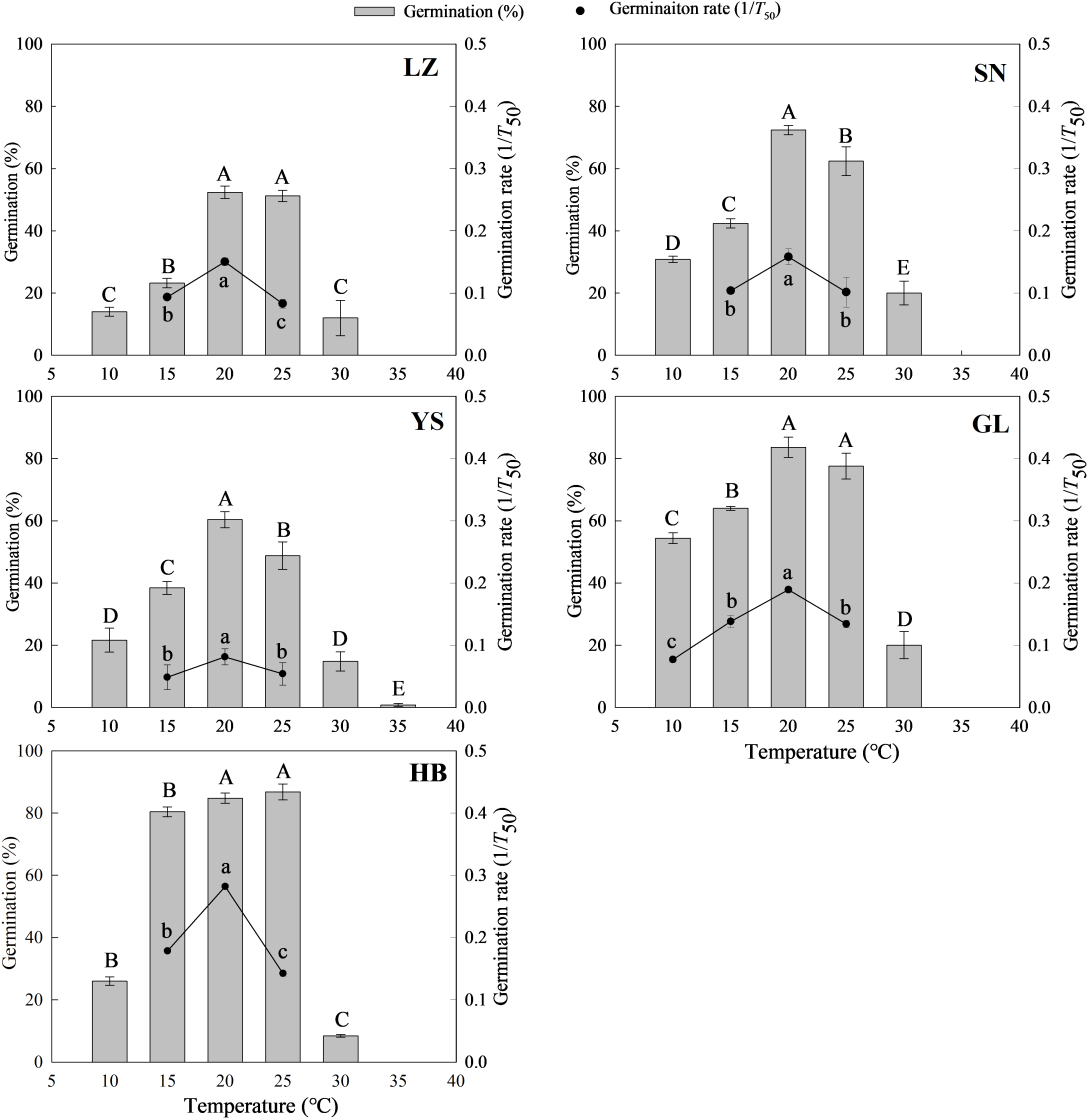
Seed germination percentage and rate (1/T50) of P. kansuensis from five populations in a temperature gradient. The different capital or lowercase letters indicate the mean significant difference (P< 0.05) for the germination percentage and rate at different temperatures.

Plant origins, temperature, and their interaction of the two had significant effects on seed germination rate (1/*T*_50_) and percentage (**Table S2**). Generally, the germination percentage and rate initially increased and then decreased with increased temperature, and a higher germination percentage was observed at 20°C or 25°C (**Figure 1**). The germination of *P*. *kansuensis* seeds from LZ was lower at the designed temperatures than that of seeds from the other four habitats (**Table S2** and **Figure 1A**). At a lower temperature (10°C), seed germination was higher for *P*. *kansuensis* from GL than from other habitats (**Table S2** and **Figure 1D**). By contrast, only the seeds from YS germinated at 35°C (**Figure 1C**).

**Figure 2:**
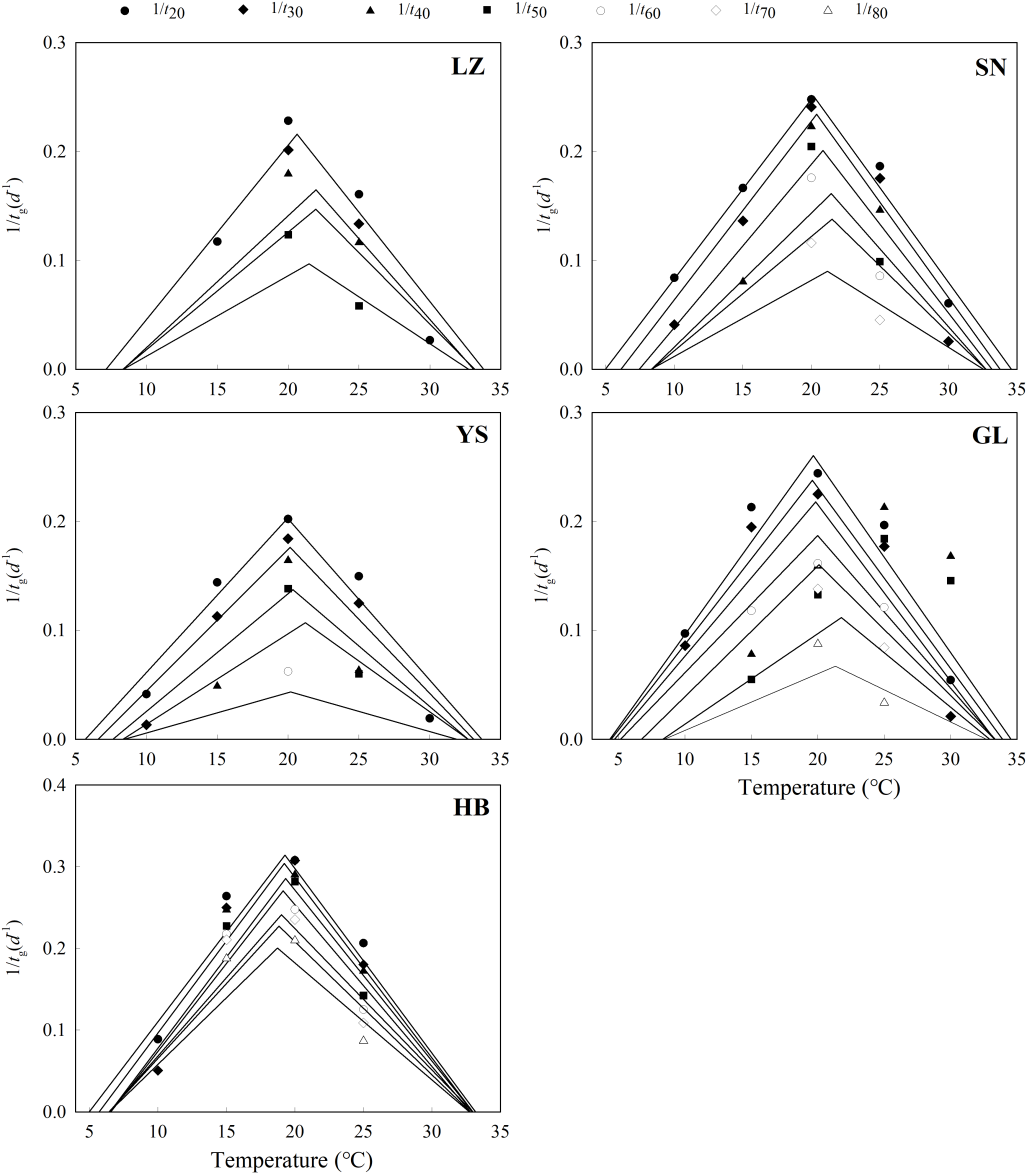
Linear regression of the germination rate (1/tg) of different percentiles and temperatures at suboptimal and supraoptimal temperature ranges of *P. kansuensis* from five populations.

**Figure 3:**
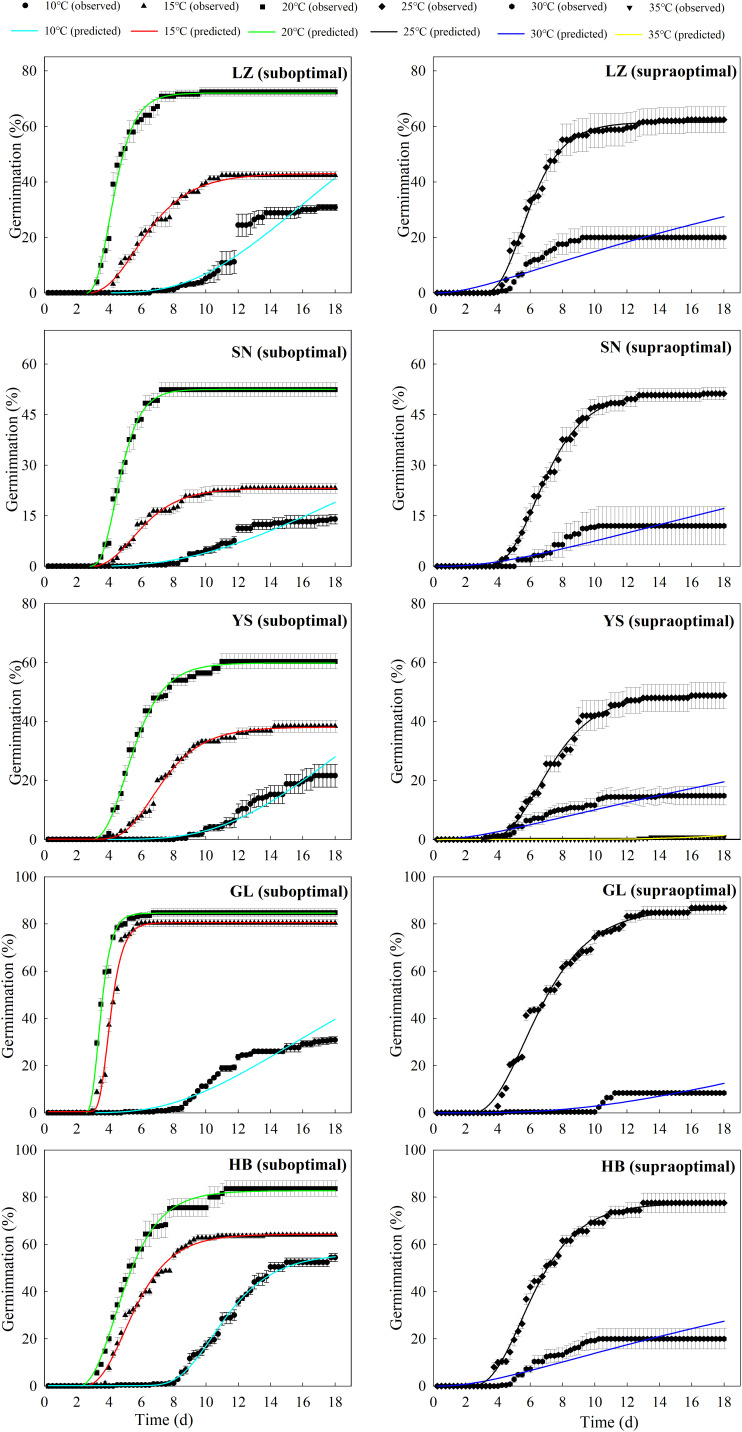
Predicted (line) and observed (dot) germination time course of the response of *P. kansuensis* from five populations to different temperatures at the suboptimal (left) and supraoptimal (right) temperature ranges.

**Figure 4:**
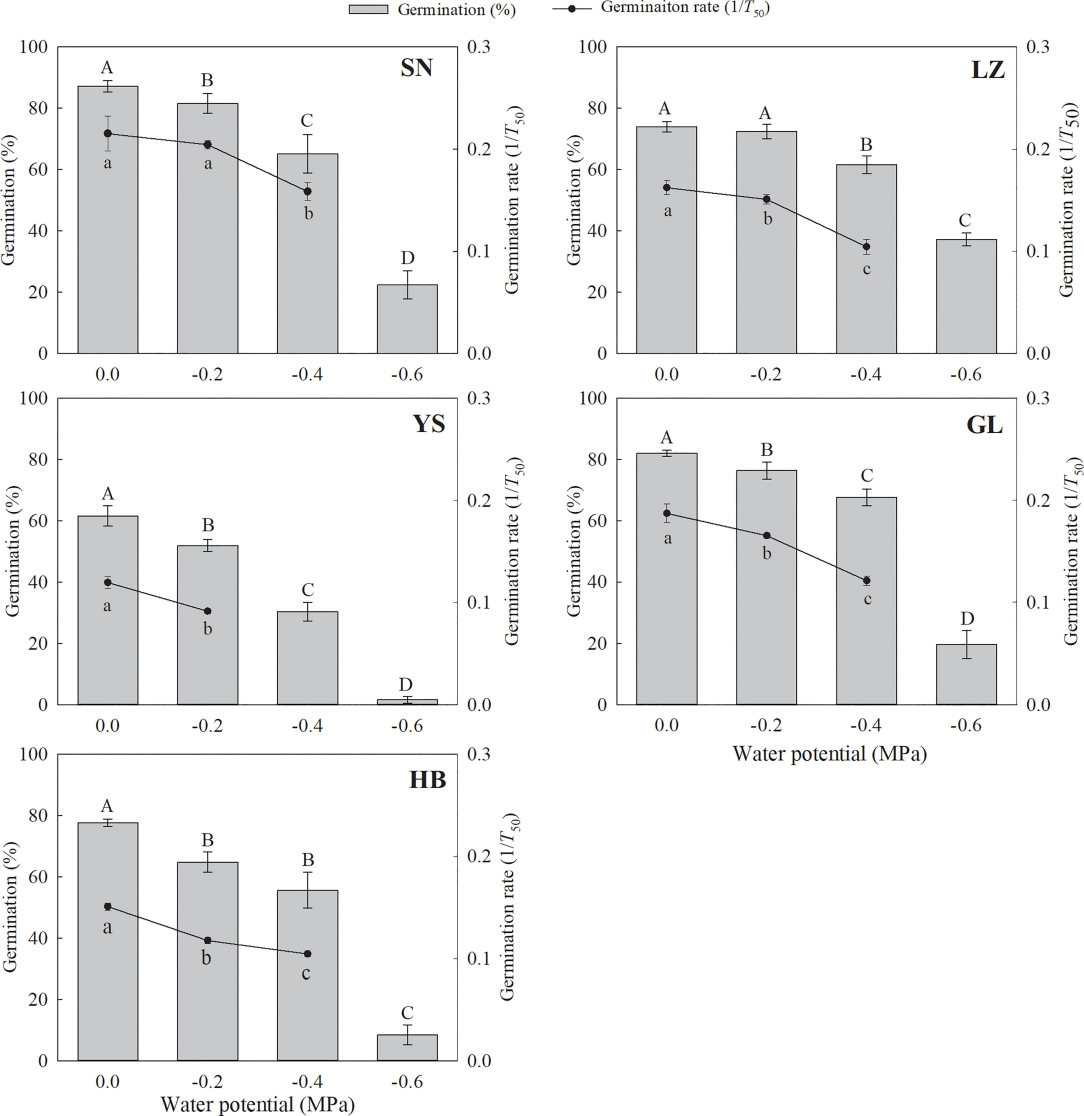
Seed germination percentage and rate (1/*T*_50_) of *P. kansuensis* from five populations at different water potentials. The different capital or lowercase letters indicate the mean significant difference (*P*< 0.05) for the germination percentage and rate at different water potentials.

The seeds from warm habitats had a relatively higher *T*_b_ than those from cool habitats (**Table 3** and **Figure 2**). According to the extrapolation model, the average *T*_b_ from warm and cool habitats was 7.7°C and 6.2°C, respectively; there was no difference in *T*_o_ and *T*_c_ between the cool and warm habitats (**Table S3** and **Figure 3**). Based on the thermal time model at suboptimal and supraoptimal temperatures, the dynamics of seed germination for all populations were well described at 15°C, 20°C, and 25°C (**Figure 4**), and the estimated value of *T*_b_ from cool habitats at suboptimal temperatures was lower than that of seeds from warm habitats (**Table 4**). This is consistent with the results derived from the extrapolation method.

**Table 3:** Estimation of the three cardinal temperatures using a linear regression of seed germination rate (1/tg) as a function of temperature in *P. kansuensis* seeds from warm and cool habitats.

Habitat	Origin	*T*_b_	*T*_o_	*T*_c_
Warm	LZ	8.0 ± 0.3a	21.5 ± 0.3a	33.2 ± 0.2a
	SN	7.3 ± 0.6ab	20.9 ± 0.2ab	33.3 ± 0.3a
Cool	YS	6.9 ± 0.5ab	20.4 ± 0.2bc	32.8 ± 0.3a
	HB	6.2 ± 0.2bc	19.1 ± 0.1d	32.9 ± 0.1a
	GL	5.6 ± 0.7c	20.1 ± 0.3c	33.6 ± 0.2a

The different lowercase letters indicate the significant differences (*P* < 0.05) in the cardinal temperatures for seed germination of *P. kansuensis* from different origins. *T*_b_ indicates the base temperature, *T*_o_ indicates the optimal temperature, and *T*_c_ indicates the ceiling temperature.

**Table 4:** Seed germination parameters for *P. kansuensis* originating from warm and cool habitats, based on a thermal–time model analysis at suboptimal and supraoptimal temperatures.

Habitat	Origin	Suboptimal temperature				Supraoptimal temperature			
		*θ*_T(50)_ (°C)	*σ*_θT_	*T*_b_ (°C)	*R* ^2^	*T*_c(50)_ (°C)	*σ*_Tc_	*θ*_T_ (°Cd)	*R* ^2^
Warm	LZ	61	4.01	8.3	0.90	32.49	5.15	79	0.96
	SN	51	3.56	7.5	0.89	32.08	5.00	61	0.92
Cool	YS	86	4.90	7.4	0.85	31.45	6.98	85	0.91
	GL	70	4.16	5.4	0.82	32.77	4.78	65	0.93
	HB	22	2.26	6.6	0.83	30.61	2.47	41	0.91

θ_T(50)_ is the thermal time for 50% of the seeds to germinate, σ_θT_ is the standard deviation of θ_T(50)_, T_b_ is the constant base temperature in the suboptimal temperature range, T_c(50)_ is the maximum temperature for 50% of the seeds to germinate, σ_Tc_ is the standard deviation of T_c(50)_, and θ_T_ is the constant thermal time.

The seed germination percentage and rate decreased significantly with a decrease in water potential (**Table S4** and **Figure 4**). The seeds originally collected from YS and HB germinated to only 8.4% and 1.6%, respectively, at −0.6 MPa. Conversely, the seeds that originated from LZ, SN, and GL germinated to 37.2%, 22.4%, and 19.6%, respectively, at −0.6 MPa.

**Figure 5:**
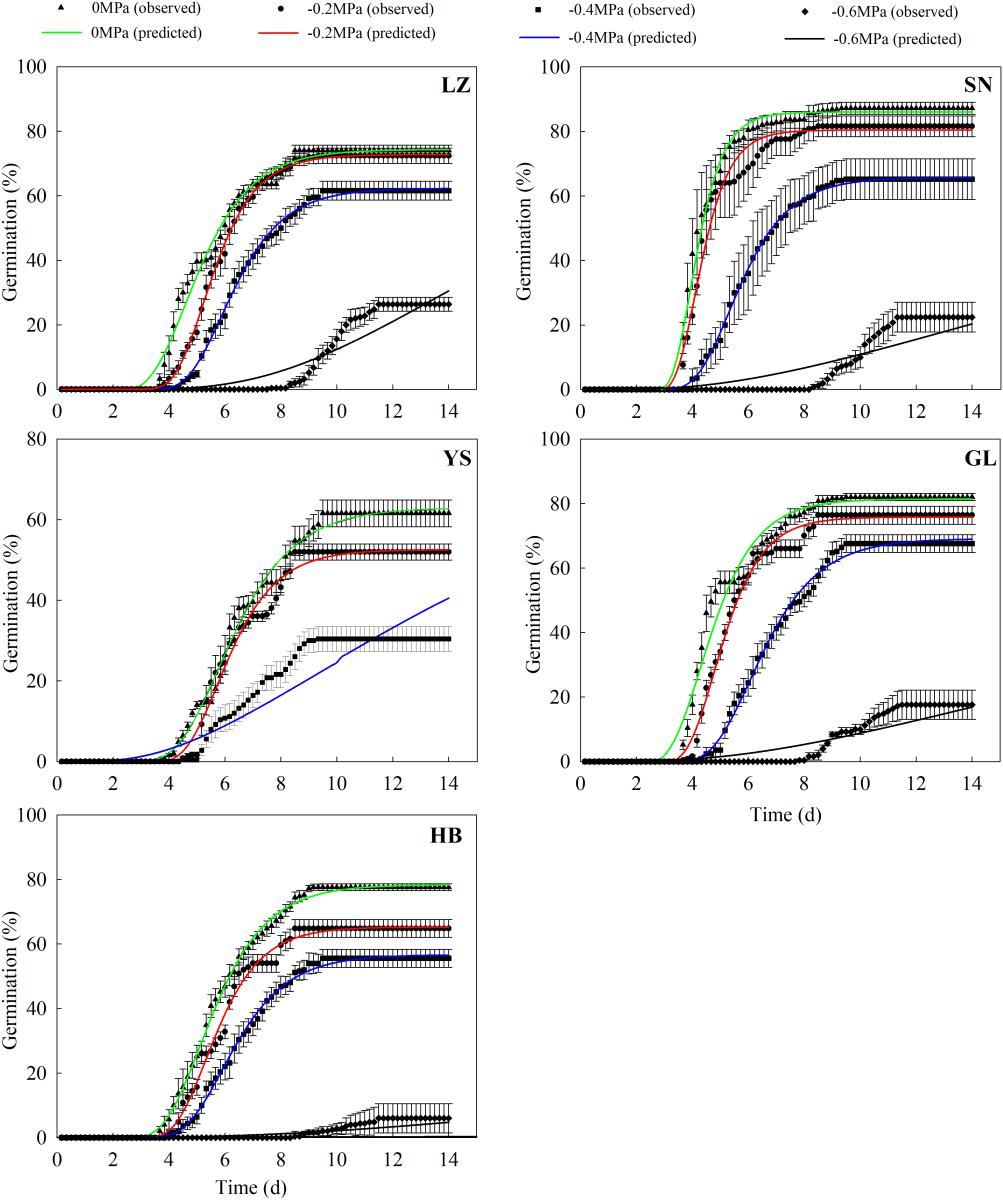
Predicted (line) and observed (dot) germination time course of the response of *P. kansuensis* from five populations to different water potentials.

The hydrotime model described *P*. *kansuensis*’s germination dynamics well in response to a high water potential (≥ −0.04 MPa, **Figure 5**). However, this model failed to demonstrate the germination process at a lower water potential (< −0.04 MPa) for different populations (**Table 5** and **Figure 5**). The seeds collected from YS had the lowest *Ψ*_b(50)_ (−1.31 MPa), whereas those from HB had the highest *Ψ*_b(50)_ (−0.68 MPa). Furthermore, the hydrotime constant (*θ*_H_) differed among the five populations (**Table 5**), with the lowest *θ*_H_ being detected in the seeds from HB (2.6 MPd) and the highest from YS (9.8 MPd). Therefore, *Ψ*_b(50)_ was negatively correlated with *θ*_H_ for the seeds originating from each of five natural habitats.

**Table 5:** Seed germination parameters for the response of the five *P. kansuensis* populations from warm and cool habitats to water potential based on the hydro–time model analysis.

Habitat	Origin	*θ*_H_ (MPa.d)	*Ψ*_b(50)_ (MPa)	σ_Ψb_	*R* ^2^
Warm	LZ	4.1	−0.84	0.26	0.74
	SN	5.4	−1.03	0.30	0.82
Cool	YS	9.8	−1.31	0.53	0.93
	GL	4.7	−0.90	0.24	0.81
	HB	2.6	−0.68	0.20	0.80

θ_H_ is the constant hydrotime, Ψ_b(50)_ is the base water potential for 50% of the seeds to germinate, and σ_Ψb_ is the standard deviation of Ψ_b(50)_.

## Discussion

The results suggest that the seed germination of *P*. *kansuensis* originating from warm habitats had a higher base temperature than that of seeds collected from cool habitats, but no difference was detected in *T*_o_ and *T*_c_ between warm and cool habitats. Furthermore, seed germination in response to water potentials differed among the five populations. A negative correlation was found between the base water potential for 50% (*Ψ*_b(50)_) germination and the hydrotime constant (*θ*_H_) in seeds from all the five natural populations.

### Germination responses to the temperature of *P*. *kansuensis* from different populations

The traits of seed germination strongly depend on the environmental conditions in which the maternal plants grow (Donohue et al., 2005). Temperature, in particular, is considered a crucial abiotic factor in determining seed germination, species distribution, and community composition (Fenner and Thompson, 2005; Cochrane et al., 2014). Species originating from tropical regions always require a higher *T*_b_ for seed germination than those from temperate regions (Trudgill et al., 2000). Dürr et al. (2015) concluded that crop species of tropical origins have the highest *T*_b_, whereas wild species and trees originating from cool growing regions have the lowest *T*_b_. Similar conclusions have been drawn for the same genus from different natural populations (Steinmaus et al., 2000; Hu et al., 2015; Bao et al., 2019; Zhang et al., 2020). For example, legume or *Stipa* species collected from the Qinghai–Tibet Plateau have a lower *T*_b_ for germination than those collected from the Alax Desert and Loess Plateau (Hu et al., 2015; Zhang et al., 2020). Consistent with these findings for the same genus, one species from cool habitats can have a lower cardinal temperature for seed germination than the same species from warm habitats. For example, Bao et al. (2019) reported that *Achnatherum inebrians* seeds from cool habitats had a lower *T*_b_, *T*_o_, and *T*_c_ for seed germination than those from warm habitats. In the present study, although the *P*. *kansuensis* seeds all originated from populations inhabiting the Qinghai–Tibet Plateau, the seeds from warm habitats (SN and LZ) had a higher *T*_b_ for germination than the seeds from cool habitats (HB, YS, and GL). By contrast, *P*. *kansuensis* from the Tianshan Mountains of Northwest China can germinate at 5°C (Hu et al., 2022). This does not conflict with the present study’s findings, as the annual average temperature of *P*. *kansuensis* originating from the Tianshan Mountains (−4.8°C) was lower than that of the populations in our study (2.1°C). Thus, none of the *P*. *kansuensis* seeds from the Qinghai–Tibet Plateau germinated at 5°C. A possible explanation for this variation is that species distributed across wide geographical regions often demonstrate relatively large variations in germination characteristics according to their provenance (Imbert et al., 1999; Fenner and Thompson, 2005). Imbert et al. (1999) and Baskin and Baskin (2014) suggest that temperature during the seed maturation of one species from different populations, has a local adaptive effect on the offspring germination characteristics of seeds (Imbert et al., 1999) because the minimum temperature required for germination provides an adaptive benefit to prevent premature or overdue germination during the Qinghai–Tibet Plateau’s short summer (Hu et al., 2015). Furthermore, the adaptive strategy of germination in response to habitat temperature also largely determines the destiny of the recruited seedlings, as seedling survival is largely enhanced after snowmelt, when germination is triggered by a relatively high temperature in the late spring or early summer (Alvarado and Bradford, 2002; Shimono and Kudo, 2005; Zhang et al., 2020).

The optimum temperature for the germination of *P*. *kansuensis* from cool habitats was slightly higher than that of the seeds from warm habitats, but the ceiling temperature showed no difference among provenances. All test seeds germinated well at a temperature interval of 20–25°C, suggesting that a low temperature during seed maturation and dispersal could prevent the emergence of seedlings and their death by freezing the following winter (Fenner and Thompson, 2005; Wesche et al., 2006). However, this study was inconsistent with other studies (Wang et al., 2008; Sui et al., 2013; Hu et al., 2022) in terms of the optimum temperature for *P*. *kansuensis* germination. Hu et al. (2022) showed that *P*. *kansuensis* germination was significantly higher at 25°C than at 20°C. The variation in the optimum temperature for germination in the studied populations of *P*. *kansuensis* may be related to genetic differentiation, environmental conditions, or the interactions of the two. The maternal plant’s genetics and environmental conditions during seed maturation and dispersal are considered crucial factors in the difference in germination requirements among the populations of a single species (Fenner and Thompson, 2005; Baskin and Baskin, 2014). Previous studies have found that annual temperature is one of the most important environmental variables determining the distribution of *P*. *kansuensis* (Sui et al., 2013; Wang et al., 2019), suggesting that the habitat occupied by this weed will continue to grow in western China due to rising temperatures (Wang et al., 2019).

### Germination responses to the water potential of different populations of *P*. *kansuensis*

Seeds adapted to germinating under water stress conditions are generally considered to have an advantage in germinating in arid or semiarid environments (Fenner and Thompson, 2005). However, there may be a trade-off between seed germination under low water potential and seedling survival after germination; seeds with higher germination at reduced water potential often have relatively low seedling establishment in arid regions, as seedlings require more water to compensate for evaporation (Flores and Briones, 2001). Previous studies have suggested that the relationship between seed germination and water stress/tolerance is largely species-specific and dependent upon environmental conditions (Fenner and Thompson, 2005; Baskin and Baskin, 2014). For example, Evans and Etherington (1990) reported that species originating from wetlands could not germinate well at low water potentials. By contrast, *Rumex crispus* from arid habitats could germinate when the water potential level was down to –1.5 MPa (Evans and Etherington, 1990). Zeng et al. (2010) found that species from semiarid regions achieved relatively higher germination levels at −1.8 MPa than those from arid habitats. Therefore, the seeds of species from humid habitats should be more sensitive to low water potentials than those from arid habitats, because the selection pressure from soil stress for seeds originating form humid habitats is lower than that of seeds originating from arid habitats (Ludewig et al., 2014).

Different populations of the same species may demonstrate different germination abilities when they germinate under water stress (Baskin and Baskin, 2014; Lima and Meiado, 2017). Previous studies found that the seed germination of *Pilosocereus catingicola*, *Betula pendulai*, and *Pinus brutia* from dry habitats was higher than that of the same plants from wet habitats with low water potentials (Lima and Meiado, 2017; Tilki and Dirik, 2007; Ranno et al., 2020). Similarly, Zhang et al. (2017) reported that the germination of *Stipa bungeana* from Western China’s Loess Plateau decreased with decreased water potential in eight populations, and that sensitivity to water stress varied among the populations. In the present study, the germination of *P*. *kansuensis* from different populations also differed when the seeds were subjected to water stress. The populations’ sensitivity to water stress is ranked as follows: HB (−0.68 MPa)< LZ (−0.84 MPa)< GL (−0.90 MPa)< SN (−1.03 MPa)< YS (−1.31 MPa), this ranking indicates that habitat had no plasticity to seed germination of *P*. *kansuensis* response to water stress. This finding is consistent with Daws et al. (2008), who detected no clear pattern in sensitivity to water potential in relation to habitat type for the germination of Neotropical species. The possible reasons for the within-population differences of *P*. *kansuensis* germination under water stress may be related to genetic differentiation and the moisture of the soil in which the maternal plants produced their seeds (Li et al., 2016). Recent studies have indicated that gene flow among *P*. *kansuensis* populations is limited to the Qinghai–Tibet Plateau (Li et al., 2016). Consequently, a high level of genetic differentiation has been shown among the populations (Li et al., 2019). Furthermore, the distance between collection sites was greater than 450 km, and the five populations likely had genetic differences. Therefore, genetic differences may explain, in part, the population differences in seed germination under low water potential (Zhang et al., 2017). Variations in soil water across seed collecting sites can also explain the differences in germination among the populations (Wang et al., 2019; Yang et al., 2020), as the annual precipitation of each habitat is significantly different. However, seed germination may be determined by other features of the maternal environment (Fenner and Thompson, 2005; Baskin and Baskin, 2014). Therefore, other environmental factors should also be considered when studying the germination characteristics of a single species across a wide geographical range.

A negative relationship has been noted between *θ*_H_ and *Ψ*_b(50)_ at a given temperature when testing the germination response at different water potentials. This suggests that seeds with a low *θ*_H_ and high *Ψ*_b(50)_ may germinate quickly under sufficient water conditions but prevented from germination under water stress conditions (Bradford, 1999; Batlla and Benech-Arnold, 2004; Zhang et al., 2020; Ullah et al., 2022). The present study supports these findings: the seeds originating from YS and SN, with high tolerance to water stress (low *Ψ*_b(50)_), spent more time germinating than those from HB and LZ, with high *Ψ*_b(50)_. This gives seeds with a high *θ*_H_ on the Qinghai–Tibet Plateau an ecological advantage because seeds germinate during the driest period of the growing season (May) in this region (Hu et al., 2015). A high *θ*_H_ would inhibit germination after a rainfall event and prevent the death of seedlings after a subsequent drought (Zhang et al., 2020).

## Conclusions

The results demonstrate that seeds from cool habitats have a higher base temperature than those from warm habitats, suggesting that seeds from Qinghai (cool habitats) are more tolerant at high temperatures than those from Tibet (warm habitats); By contrast, there is no clear pattern in sensitivity to water potential in relation to habitat type, indicating that the temperature requirements (rather than the water potential) for the seed germination of *P*. *kansuensis* originating from different populations are closely related to environmental conditions and habitats. Furthermore, both thermal and hydrotime models are good predictors of seed germination course for the non-dormant seeds of five *P*. *kansuensis* populations in response to temperature and water potential on the Qinghai–Tibet Plateau, and these models can be employed to predict the distribution and spread of this hemiparasitic weed across alpine regions in climate change scenarios. However, these results should be interpreted cautiously because only five *P*. *kansuensis* populations and two habitats have been included in our study. Future studies should expand the sampling sites for a comprehensive understanding of the potential effect of maternal habitat microclimate on this hemiparasitic weed’s seed germination, seedling establishment, and population distribution and expansion in alpine regions.

## Data availability statement

The original contributions presented in the study are included in the article/**Supplementary Material**. Further inquiries can be directed to the corresponding author.

## Author contributions

GB designed and conducted this study. PZ and XW collected seed materials and conducted this study. YZ and WL provided some critical suggestions about the manuscript. GB and PZ wrote the manuscript together. All authors contributed to the article and approved the submitted version.

## Acknowledgments

We would like to thank associate Professor Yong Liu for his valuable comments on the manuscript. This study was financially supported by the basic research program of science and technology of Qinghai Province, China (2022-ZJ-715) and the Natural Science Foundation of China (Grants 32060398 and U21A20239).

## Conflict of interest

The authors declare that the research was conducted in the absence of any commercial or financial relationships that could be construed as a potential conflict of interest.

## Publisher’s note

All claims expressed in this article are solely those of the authors and do not necessarily represent those of their affiliated organizations, or those of the publisher, the editors and the reviewers. Any product that may be evaluated in this article, or claim that may be made by its manufacturer, is not guaranteed or endorsed by the publisher.
