# Supplementary material for: Comparison of the effect of temperature and water potential on the seed germination of five Pedicularis kansuensis populations from the Qinghai–Tibet plateau
Source: Front Plant Sci. 2022 Nov 24;13:1052954. doi: 10.3389/fpls.2022.1052954 (PMC9731731; doi:10.3389/fpls.2022.1052954)
Supplement: Supplementary file 1 [file DataSheet_1.docx]

**Supporting Information**

**This file includes:**

Tables S1-S4

**Table S1** One-way analysis of variance (ANOVA) results for seed size and thousand seed weight of *P. kansuensis* seeds originating from different populations

| Source of variation | df | Length | |  | Width | |  | Thousand seed weight | |
| --- | --- | --- | --- | --- | --- | --- | --- | --- | --- |
|  |  | *F* | *P* |  | *F* | *P* |  | *F* | *P* |
| Origin | 4 | 31.77 | < 0.01 |  | 1.08 | 0.37 |  | 177.96 | < 0.01 |

**Table S2** Two-way ANOVA results for the seed germination percentage and rate (1/*T*_50_) of five *P. kansuensis* seed populations under different temperature regimes

| Source of variation | df | Germination percentage | |  | Germination rate (1/*T*_50_) | |
| --- | --- | --- | --- | --- | --- | --- |
|  |  | *F* | *P* |  | *F* | *P* |
| Origin | 4 | 26.56 | < 0.01 |  | 47.43 | < 0.01 |
| Temperature | 5 | 826.19 | < 0.01 |  | 164.33 | < 0.01 |
| Origin × Temperature | 20 | 5.14 | < 0.01 |  | 16.85 | < 0.01 |

**Table S3** One-way ANOVA results for the cardinal temperatures (*T*_b_, *T*_o_ and *T*_c_) of *P. kansuensis* seeds collected from five populations based on a linear regression analysis

| Source of variation | df | *T*_b_ | |  | *T*_o_ | |  | *T*_c_ | |
| --- | --- | --- | --- | --- | --- | --- | --- | --- | --- |
|  |  | *F* | *P* |  | *F* | *P* |  | *F* | *P* |
| Origin | 4 | 3.67 | 0.02 |  | 2.54 | 0.07 |  | 1.68 | 0.19 |

**Table S4** Two-way ANOVA results for the seed germination percentage and rate (1/*T*_50_) of five *Pedicularis kansuensis* seed populations under different water potentials

| Source of variation | df | Germination percentage | |  | Germination rate (1/*T*_50_) | |
| --- | --- | --- | --- | --- | --- | --- |
|  |  | *F* | *P* |  | *F* | *P* |
| Origin | 4 | 34.02 | < 0.01 |  | 17.67 | < 0.01 |
| Water potential | 3 | 158.46 | < 0.01 |  | 105.64 | < 0.01 |
| Origin × Water potential | 20 | 11.97 | < 0.01 |  | 2.98 | < 0.01 |
